# Raising Stroke Prevention Champions: Evaluation of a Nurse-Led Primary Health Care Nurse Training Intervention

**DOI:** 10.3390/nursrep16020037

**Published:** 2026-01-23

**Authors:** Mpho Z. Shelile, Bokang A. Mahlelehlele, Nick Bass

**Affiliations:** 1 Department of Nursing, Faculty of Health Sciences, National University of Lesotho, Maseru P.O. Roma 180, Lesotho; ba.mahlelehlele@nul.ls; 2 Global Health, East London Foundation Trust, National Health Service, East London, London E15 4PT, UK; nick.bass@nhs.net

**Keywords:** primary health care, registered nurse, stroke prevention champion, stroke

## Abstract

**Background**: Globally, stroke is a significant health problem and is considered one of the leading causes of mortality and permanent disability worldwide. Nurses are key stakeholders and integral members of the stroke care team, contributing to every stage of care. **Objective**: This study aimed to assess the effects of a nurse-led stroke training program on the knowledge, attitudes, and practices of registered nurses working in Berea primary health care facilities, Lesotho, before and after an educational intervention. **Methods**: To evaluate the effectiveness of the nurse-led intervention, this study used a pre-experimental one-group pretest–posttest design. Participants completed a structured questionnaire before the intervention to assess their baseline knowledge, attitudes, and practices related to stroke. Following the intervention, which consisted of educational sessions led by trained nurses, the same questionnaire was administered again to measure changes in participants’ knowledge, attitudes, and practices. Paired *t*-tests compared results. **Results**: A total of 34 registered nurses from 18 primary healthcare facilities participated in this pre- and post-intervention questionnaire study. When comparing knowledge, attitudes, and practices related to stroke before and after the educational intervention, the increase in correct response rates was statistically significant (*p* < 0.001). The training evaluation received positive feedback from the participants. **Conclusions**: Structured nurse-led educational interventions enhance nurses’ knowledge, attitudes, and practices in stroke care, leading to improved patient outcomes and stronger community-based prevention. These findings highlight the need to integrate continuous stroke education into nursing policies and primary health care practice.

## 1. Introduction

A stroke, or cerebrovascular accident, is an acute disruption of cerebral blood flow, and about 85% of strokes are ischemic, with the remainder hemorrhagic [1]. Stroke has emerged as a significant public health concern, ranking among the leading causes of death and disability worldwide [2]. The World Health Organization estimated that in 2005 there were approximately 16 million new stroke cases and 62 million survivors, with stroke-related deaths representing 9.7% of all global deaths [2]. Without a strong global public health response, these numbers are projected to rise to more than 23 million new cases and 7.8 million deaths by 2030 [2]. Recent global estimates also highlight that stroke remains one of the largest contributors to death and disability worldwide [3]. Current syntheses report that about 15 million people experience a stroke each year, with roughly 5 million deaths and another 5 million people left permanently disabled, and global stroke disability-adjusted life years exceeding 160 million, making stroke a leading cause of death and disability globally [4].

The burden of stroke is shifting; reductions seen in some high-income settings have plateaued or reversed in many low- and middle-income regions [5]. Evidence further indicates that the Middle East faces a double burden, as the prevalence of stroke coincides with increasing rates of non-communicable diseases [6]. Rates of stroke incidence, mortality, and disability are rising fastest in low- and middle-income countries, including regions of sub-Saharan Africa, driven largely by uncontrolled hypertension and other cardiovascular risk factors [7]. This rising trend threatens to increase both human suffering and economic costs unless prevention and treatment capacity are urgently strengthened.

Lesotho’s health care system is heavily nurse-driven, particularly at the primary care level, where registered nurses and nurse clinicians are the backbone of service delivery [8]. Nurses make up the largest cadre in the health workforce, accounting for about 33% of all health personnel and 90% of those directly delivering care [9]. Primary health care (PHC) in Lesotho is organized through a tiered system that includes village-level health workers, clinics/health centers, and Health Service Areas, each anchored by a hospital but staffed largely by nurses and nurse practitioners [10]. Lesotho’s health service, delivered at multiple levels (village health workers, clinics, health-service areas, district hospitals), faces a double burden of disease, with high rates of communicable illnesses such as HIV/AIDS, tuberculosis (TB), and respiratory infections, alongside a growing prevalence of non-communicable diseases (NCDs) such as hypertension, diabetes, cardiovascular disease, and trauma [8]. Obesity, closely linked to these conditions, is rising sharply, particularly among middle-aged adults, the wealthy, and those with higher education [6]. These disparities, coupled with aging and lifestyle changes, increase vulnerability to hypertension, diabetes, and cardiovascular disease, thereby elevating the risk of stroke. Stroke is already a major health threat in Lesotho, accounting for 2817 deaths in 2020 (8.45% of total deaths), with an age-adjusted death rate of 211.66 per 100,000, ranking Lesotho third highest globally [8]. Because cardiovascular risk factors (hypertension, diabetes, overweight) are highly prevalent across age groups in Lesotho, younger and middle-aged adults are increasingly vulnerable [11]. This means loss of employment and income potential are high; survivors of stroke often face significant disability, reduced capacity for work or needing to retire early, placing a large financial strain on households. The economic impact of stroke includes both direct health-care costs and indirect losses in productivity and potential long-term disability, all of which hamper national economic growth, particularly in a lower-income context like Lesotho’s [12]. Furthermore, all United Nations member states have pledged to achieve the Sustainable Development Goals (SDGs), yet only a few are currently on track to meet SDG 3.4, which aims to reduce premature mortality from non-communicable diseases, including stroke, by one-third through prevention and treatment, and to promote mental health and wellbeing by 2030 [4]. This underscores the urgent need for community-based stroke prevention and management interventions.

Stroke is considered the most preventable neurological disorder due to its well-established risk factors. Findings from the INTERSTROKE studies indicate that hypertension, high cholesterol, diabetes, smoking, obesity, physical inactivity, unhealthy diets, and excessive alcohol consumption are the leading contributors to stroke worldwide [13,14,15]. Importantly, these factors can largely be mitigated through healthier lifestyle practices and positive behavioral changes.

Research has demonstrated that limited awareness of the leading contributors to stroke and warning signs contributes significantly to delays in seeking hospital care, which in turn may lead to poorer outcomes [16]. Enhancing public knowledge of these risk factors and warning signs is therefore essential in controlling the disease and reducing both morbidity and mortality associated with stroke. One of the primary goals of nursing, accordingly, is to promote stroke awareness among high-risk populations and to assess their understanding of the consequences of their conditions in relation to stroke [17].

In stroke management, healthcare professionals involved in stroke care must demonstrate a strong commitment to service, possess relevant expertise, and exhibit effective communication skills. Furthermore, ongoing education and training should be provided to staff who lack adequate knowledge or skills [18]. Nurses serve as key stakeholders and essential members of the multidisciplinary stroke care team, contributing to every stage of care—from initial assessment and symptom recognition to treatment, rehabilitation exercises, early warning monitoring, psychological support, and end-of-life care [19,20]. Therefore, stroke nurses need comprehensive education and training to enhance their ability to provide high-quality care for stroke patients. Developing and implementing stroke education and training programs for nurses is essential to ensuring quality stroke care and improving patient outcomes [21].

Education also plays a vital role in stroke recovery for both patients and their families [22]. The complexity of stroke-related impairments and the life adjustments they necessitate often result in diverse and unmet educational needs. Patients and caregivers frequently seek information about stroke pathophysiology, prevention, treatment options, functional recovery, patient mobility, exercise routines, psychological changes, and nutritional guidance [23]. When these educational needs are not addressed, they may lead to anxiety, emotional distress, misinformed decisions, and poor health outcomes. Thus, providing comprehensive and tailored education is essential for supporting stroke survivors and their families throughout the recovery journey [24].

The Medical Research Council highlights the critical importance of evaluating both the feasibility and the implementation of health interventions, such as educational programs [25]. This evaluation should consider multiple factors, including the acceptance of the program by healthcare professionals, the current knowledge and skill levels of nursing staff, and the availability of resources and facilities required for successful implementation [26,27]. Such a comprehensive assessment ensures that educational interventions are not only theoretically sound but also practical and sustainable within the healthcare setting [26]. In line with this perspective, this paper presents the results of Phase 2 of a four-phase nurse-led, “Phela u Phelise” community-based stroke prevention intervention. The overall goal of this community-based nurse-led intervention was to prevent, manage, and reduce the impact of stroke by promoting self-management of predisposing factors and improving access to high-quality stroke nursing care. This paper presents the results of Phase 2, in which nurses designated as Stroke Prevention Champions (SPCs) were capacitated and supported to deliver high-value care, promote lifestyle interventions, and strengthen their ability to mentor and guide village health workers. Although nurse-led health promotion initiatives are well established globally, this intervention is relatively new in the context of community-based stroke prevention in Lesotho. The phase was intentionally structured to respond to gaps in nurses’ knowledge, attitudes, and clinical practices related to stroke care. This phase focused on strengthening nurses’ knowledge, fostering positive attitudes, and improving clinical practices related to stroke care. The educational intervention included structured training on stroke risk factors, early recognition of symptoms, emergency response, secondary prevention strategies, and patient and family education. By evaluating participants’ knowledge, attitudes, and practices before and after the intervention, this phase aimed to determine the intervention’s effectiveness and its potential to build long-term nursing capacity for stroke prevention and management in community settings.

## 2. Materials and Methods

This study used a pre-experimental one-group pretest–posttest design. A pretest–posttest study was conducted in March 2025 to evaluate the effects of an educational intervention on registered nurses’ knowledge, attitudes, and clinical practices concerning stroke care.

The workshop was specifically designed to equip registered nurses with the knowledge and skills necessary to act as Stroke Prevention Champions (SPCs) within their respective primary healthcare facilities, thereby improving stroke prevention, recognition, and care at the community level.

The study involved 34 registered nurses from 18 primary healthcare facilities in the Berea District. Recruitment involved sending formal letters to managers of primary healthcare facilities in the Berea District, requesting that each facility release two registered nurses with at least six months of clinical experience to attend a five-day training program. The training, which included the pre-test, educational sessions, and post-test assessment, was conducted at a designated venue. Of the 18 facilities, two sent only one nurse instead of two, resulting in a total of 34 participants. The selected registered nurses were required to demonstrate a willingness to participate in the training program, which aimed to equip them with the knowledge and skills to become Stroke Prevention Champions. In addition to enhancing their clinical competencies, these nurses were expected to serve as trainers for village health workers and to implement community-based stroke awareness and health promotion activities, thereby extending the impact of the program beyond the healthcare facilities and into the communities they serve. This four-phased nurse-led, community-based stroke prevention intervention was approved by the Ministry of Health, Lesotho’s (ID288-2024) National Health Research and Ethics Committee. Prior to participation, all nurses received detailed information about the project, study and provided informed consent. To ensure privacy and confidentiality, the questionnaire avoided collecting personal identifiers, such as participants’ names, contact details, or the names of their facilities. Participants were also assured that the study findings would be reported anonymously.

A formal training program on the symptoms and management of acute stroke, nursing care of stroke survivors, control of vascular risk factors, and caregiver-based rehabilitation was developed for registered nurses. Table 1 shows the content covered during the training.

The training was designed and delivered by adult health nurses, community health nurses, nurse-midwives, psychiatric nurses, pharmacists, nutritionists, and environmental health specialists from the National University of Lesotho. The authors then conducted a five-day educational intervention through face-to-face lectures, using PowerPoint presentations to support the educational guide, with each presentation lasting between 40 and 60 min. The intervention was intentionally structured to address documented gaps in nurses’ knowledge, attitudes, and clinical practices related to stroke care. The content was developed using evidence from international stroke guidelines, including those from the World Health Organization [28] and the American Heart Association/American Stroke Association [29], and was further informed by empirical studies demonstrating the effectiveness of nurse-led stroke education in improving early recognition, treatment adherence, and community-level prevention outcomes [30,31].

Prior to implementation, the educational tools and training materials were piloted with a small group of nurse educators and practising nurses to assess clarity, relevance, and feasibility. Feedback from this pilot informed refinements to the training manuals, case scenarios, and assessment tools, consistent with recommendations for intervention development in low-resource settings [32]. Although direct patient or carer involvement, particularly individuals with lived experience of stroke, was not incorporated into this phase, this was largely due to resource constraints, limited availability of organized stroke survivor groups, and logistical challenges in engaging patients at the community level. However, the content indirectly reflects patient perspectives through evidence drawn from global literature emphasizing the importance of patient education, caregiver burden, and the lived experience of stroke survivors in shaping effective prevention strategies [33].

The intervention was delivered as a short-course, non-accredited continuing professional development program by adult health nurses, community health nurses, nurse-midwives, psychiatric nurses, pharmacists, nutritionists, and environmental health specialists from the National University of Lesotho, rather than a formal university-accredited qualification. Funding was provided through the research grant supporting the broader four-phase project, which covered training materials, facilitation costs, and logistical support. This approach ensured that the intervention remained low-cost, scalable, and adaptable to routine practice within Lesotho’s primary healthcare system. Collectively, the design, evidence base, piloting process, and delivery mechanisms contribute to a robust, contextually grounded intervention aimed at strengthening nursing capacity for stroke prevention and management.

The developed assessment tool was divided into three sections: knowledge, attitude, and practice. The knowledge section consisted of 11 multiple-choice questions, the attitude section included 6 multiple-choice questions, and the practice section contained 5 multiple-choice questions. Prior to the commencement of the workshop, participants were provided with a structured questionnaire, hereafter referred to as the pre-test. Before distributing the pre-test, a brief overview of the study’s objectives, purpose, and procedures was presented to ensure participants’ understanding and voluntary participation. Participants were instructed to answer the questions independently, and on average, it took approximately 30 min to complete the pre-test.

Following the completion of the educational intervention, the same questionnaire was administered as a post-test immediately to evaluate any changes in participants’ knowledge, attitudes, or practices attributable to the training. Participants were again given sufficient time, averaging 30 min, to complete the post-test, after which the questionnaires were collected for analysis.

The collected data were carefully entered into a Microsoft Excel spreadsheet for initial organization and evaluation. To determine the impact of the training program, the pre-test and post-test scores of registered nurses were compared using a paired *t*-test, which is appropriate for assessing differences in means within the same group. All results were documented in Excel, and subsequent statistical analyses, including the calculation of t-values and corresponding *p*-values, were performed using GraphPad InStat 3.0 software. A *p*-value of less than 0.05 was considered indicative of statistical significance, reflecting a meaningful change in participants’ knowledge, attitudes, and practices following the intervention. Cohen’s *d* was calculated to estimate the magnitude of the training effect on participants’ knowledge, attitudes, and practices.

## 3. Results

The study questionnaire was sent to all 34 registered nurses. All the questionnaires were completed and returned, resulting in a response rate of 100%. The demographic details of the participants, along with baseline characteristics, are summarized in Table 1.

The majority (65%) of the participants were female, while the remaining (35%) were male. More than half of the participants (53%) were in the age group of 31 to 40 years, followed by 35% in the age group of 41 to 50 years, with the age groups of 26 to 30 and 51 and above years being the lowest at 2% each. Furthermore, 18% were degree nurse-midwives, and the remaining 82% were diploma nurse-midwives. The participants were of varying working experience. Table 2 depicts knowledge, attitudes and practice analysis before and after an educational intervention.

### 3.1. Knowledge, Attitudes and Practice Analysis Before and After an Educational Intervention

Table 3 illustrates the results of the assessment of knowledge, attitudes, and practices before and after the educational intervention. Knowledge, attitudes, and practice (KAP) outcomes demonstrated significant improvements across all assessed domains following the educational intervention.

#### 3.1.1. Knowledge Analysis Before and After an Educational Intervention

Regarding the strongest independent risk factor for ischemic stroke, there was a statistically significant difference between pre- and post-test responses (*p* < 0.0001). Similarly, knowledge of the acronym FAST, used for stroke screening, improved markedly, with the correct response rate increasing from 0% at baseline to 93.3% after the intervention (*p* < 0.0001).

When asked about the appropriate nursing intervention after sudden onset of unilateral weakness and slurred speech, the proportion of correct answers rose significantly from 34.1% in the pre-test to 92.5% in the post-test (*p* < 0.0001). Knowledge of the difference between ischemic and hemorrhagic stroke also improved substantially, with correct responses increasing from 8.3% before the intervention to 100% afterwards (*p* < 0.0001). Similarly, awareness of the use of intravenous thrombolysis in the management of ischemic stroke increased significantly following the training (*p* < 0.0001).

Participants also demonstrated a better understanding of diagnostic testing, with correct identification of the initial test to differentiate between ischemic and hemorrhagic stroke increasing from 8.3% in the pre-test to 96.6% in the post-test (*p* < 0.0001). In addition, knowledge of the cardiac condition most strongly associated with embolic stroke improved significantly, rising from 8.3% to 100% after the intervention (*p* < 0.0001).

Further, significant gains were observed in knowledge regarding the management of hemorrhagic stroke, with post-test responses showing marked improvement compared to baseline (*p* < 0.0001). Similarly, understanding of the most common site of cerebral infarction in ischemic stroke increased from 66.6% correct at baseline to 100% following the intervention (*p* < 0.0001).

Finally, knowledge of complications in acute stroke care also improved. Correct identification of the electrolyte imbalance that may worsen cerebral edema rose from 8.3% pre-test to 100% post-test (*p* < 0.0001). Likewise, recognition of the National Institutes of Health Stroke Scale (NIHSS) as the standard clinical tool for assessing stroke severity in emergency settings improved significantly, with correct responses increasing from 5% to 100% after the intervention (*p* < 0.0001).

#### 3.1.2. Attitude Analysis Before and After an Educational Intervention

For question twelve, which assessed responsibility for recognition and rapid referral of stroke patients, the proportion of participants indicating “a shared responsibility between nurses and the healthcare team” increased from 90% in the pre-test to 100% post-intervention (*p* < 0.0001). Question thirteen explored participants’ immediate attitude toward sudden facial drooping and slurred speech. At baseline, 73.3% of registered nurses recognized this as a medical emergency, which increased to 93.3% following the educational session.

Attitude regarding the timing of stroke rehabilitation (Question fourteen) improved substantially, with correct responses—“as early as possible during hospital admission”—rising from 8.3% in the pre-test to 100% post-test (*p* < 0.0001). Similarly, participants’ perception of their professional duty to educate stroke patients and their families about lifestyle modification (Question fifteen) increased from 20.8% pre-intervention to 100% post-intervention.

For question sixteen, assessing beliefs about stroke preventability through risk factor control, the proportion of participants responding “yes” increased significantly from 8.3% at baseline to 96.9% post-intervention (*p* < 0.0007). Finally, question seventeen examined whether nurses considered it their professional responsibility to provide training for village health workers on stroke recognition and prevention. Correct responses increased from 66.6% pre-intervention to 100% post-intervention.

#### 3.1.3. Practice Analysis Before and After an Educational Intervention

Question eighteen assessed participants’ knowledge of the priority nursing intervention upon admission of a patient with acute stroke. None of the registered nurses identified “establishing airway patency and monitoring vital signs” in the pre-test, whereas 100% responded correctly following the educational session. For question nineteen, which focused on measures before allowing oral intake in a stroke patient, 73.7% of participants indicated performing a swallowing assessment at baseline. This proportion increased significantly to 93.3% post-intervention (*p* < 0.0001).

Knowledge of deep vein thrombosis (DVT) prevention in immobile stroke patients (question twenty) also improved significantly. Correct responses—“encourage early mobilization and use compression stockings if available”—increased from 20.8% in the pre-test to 100% post-intervention (*p* < 0.0001). Similarly, when asked about secondary prevention for stroke survivors (question twenty-one), only 8.3% of participants initially indicated focusing on early mobilization and compression stockings, compared to 96.6% after training.

Finally, question twenty-two assessed the nurses’ understanding of their role in providing comprehensive stroke care. At baseline, 66.6% of registered nurses recognized that it involves acute management, rehabilitation support, and community health education. This proportion increased to 98.3% after the intervention, demonstrating a significant improvement in knowledge and understanding of holistic stroke care.

The statistical analysis shows highly significant improvements across all knowledge, attitude, and practice items (all *p* = 0.0001), indicating that the intervention had a measurable impact on participants’ learning outcomes. Using Cohen’s *d* to estimate the magnitude of change, the intervention had a very large effect on nurses’ knowledge (*d* = 2.35), attitudes (*d* = 2.51), and practices (*d* = 2.99) related to stroke care (Table 4).

## 4. Discussion

The findings of this study demonstrate that the educational intervention significantly enhanced registered nurses’ knowledge, attitudes, and practices concerning stroke care, underscoring the value of structured training programs in improving stroke management competencies. Post-intervention assessments revealed marked improvements across all domains, with many indicators approaching near-optimal levels. Despite these gains, a persistent challenge in stroke education remains translating acquired theoretical knowledge into consistent clinical practice. Evidence suggests that incorporating multiple teaching strategies, such as lectures, simulations, and practical demonstrations, can enhance nurses’ performance and the quality of patient care [34,35].

Knowledge gains were particularly notable across key areas of stroke care, including the identification of risk factors, recognition of stroke symptoms, understanding of diagnostic procedures, and implementation of management strategies. Awareness of the FAST acronym for stroke recognition, for example, increased from 0% at baseline to 93.3% post-intervention, highlighting the effectiveness of targeted education in improving early recognition skills. This aligns with previous studies showing that structured educational programs can significantly enhance nurses’ theoretical and practical knowledge, as well as their readiness to respond to acute stroke emergencies [36]. Knowledge of acute interventions; including airway management, intravenous thrombolysis, and differentiation between ischemic and hemorrhagic stroke, also improved substantially, reinforcing the importance of comprehensive training for clinical competence.

These improvements are consistent with prior research indicating that educational interventions can enhance the performance of nurses in stroke care. For instance, studies in the United States and other contexts have demonstrated that competency-based programs and lecture- plus simulation-based training improve nurses’ ability to recognize stroke symptoms and initiate timely treatment [37,38,39]. Reviews of multiple studies further emphasize that barriers to effective stroke care, such as delayed thrombolytic therapy, often stem from insufficient training and limited knowledge among healthcare staff [40,41,42]. Accordingly, educational programs that reflect the multidisciplinary nature of stroke care may be more effective in improving patient outcomes than interventions targeting only individual professional groups.

The intervention also positively influenced nurses’ attitudes toward stroke care. Participants increasingly recognized the shared responsibility of nurses and other healthcare professionals in stroke recognition and referral, and their perception of the importance of early rehabilitation and patient education improved significantly. Notably, the proportion of nurses acknowledging their responsibility to train community health workers increased from 66.6% to 100%, indicating that the program fostered professional accountability and advocacy for community-based stroke prevention. Positive shifts in attitude are critical, as they have been linked to improved adherence to clinical guidelines and better patient outcomes [43,44,45].

Knowledge and attitude gains translated into improved reported clinical practices. Post-intervention, all participants correctly identified priority nursing interventions for acute stroke, including airway management and monitoring vital signs. Increases were also observed in preventive practices, such as performing swallowing assessments before oral intake, implementing DVT prevention strategies, and applying secondary prevention measures. Additionally, understanding of holistic stroke care; including acute management, rehabilitation, and community health education; improved from 66.6% to 98.3%, highlighting the intervention’s role in strengthening the integration of comprehensive care into routine practice. These findings are supported by evidence demonstrating a positive correlation between nurses’ knowledge and clinical performance in stroke care [17,22,23]. Furthermore, educational programs targeting nurses have been shown to enhance adherence to stroke guidelines and optimize the prompt management of hospitalized stroke patients.

The baseline scores, including instances where knowledge or skills were as low as 0%, are indeed concerning, particularly given the high burden of stroke and the central role that nurses play in stroke prevention and management. Such low scores highlight critical gaps in foundational knowledge and competencies that are essential for effective stroke care. Overall, this study reinforces the importance of comprehensive stroke pre-service education and structured educational interventions in improving the KAP of nurses toward stroke care. By enhancing knowledge, fostering positive professional attitudes, and reinforcing evidence-based clinical practices, structured educational programs can contribute to better patient outcomes, timely stroke recognition, and more effective multidisciplinary care. A key strength of this study is its structured, evidence-informed nurse-led educational intervention, which demonstrated immediate improvements in nurses’ knowledge, attitudes, and self-reported practices following the five-day training. The use of pre- and post-intervention assessments allowed for a clear measurement of short-term learning gains, and the pilot testing of training materials enhanced the clarity and feasibility of the program.

## 5. Limitations

The study relied on self-reported data to assess clinical practice, which may not accurately reflect actual nursing performance. The evaluation relied primarily on immediate post-intervention assessments, which do not capture whether improvements were sustained over time. The study did not include direct observation of nurses’ clinical practice, limiting the ability to determine whether learned skills translated into real-world behavior. The absence of patient or carer involvement may have reduced the contextual relevance of some training components. Ongoing efforts are needed to ensure that improvements in theoretical knowledge are consistently translated into practical competence in real-world clinical settings. With only 34 participants, the results may not fully represent the broader population of nurses in Lesotho, whose experiences, competencies, and training needs may vary across districts. While the findings provide valuable insights into the knowledge, attitudes, and practices of the participating nurses, the limited sample size means that caution should be exercised when interpreting the results as reflective of all nurses in the country. Lastly, the findings highlight the need for a longitudinal follow-up study to assess long-term retention, practice change, and the broader impact on community-level stroke prevention outcomes.

## 6. Conclusions

Overall, this study demonstrates that structured educational interventions significantly improve registered nurses’ knowledge, attitudes, and practices regarding stroke care. By strengthening both clinical competencies and professional attitudes, such interventions have the potential to enhance patient outcomes and support broader community-based stroke prevention initiatives.

## 7. Recommendations

The results of this study have important implications for nursing practice and health system strengthening. Firstly, if utilized, educational interventions can effectively address gaps in stroke-related knowledge and practice, particularly in settings where nurses play a central role in acute and community-based care. Secondly, enhancing nurses’ attitudes toward professional responsibility and patient education may contribute to more proactive, patient-centered care. Finally, scaling up such interventions could improve early recognition, timely referral, and comprehensive management of stroke patients, potentially reducing morbidity and mortality associated with stroke.

## Figures and Tables

**Table 1:** Training program.

Session	Theme	Content
Day 1	Stroke Epidemiology and Risk Factors	○ Global, regional, and national stroke burden ○ Modifiable and non-modifiable risk factors ○ Prevention strategies in primary care
Day 2	Stroke Recognition and Acute Management	○ Clinical signs and symptoms (including FAST assessment) ○ Emergency response and referral pathways ○ Early interventions and stabilization in PHC
Day 3	Secondary Prevention and Rehabilitation	○ Blood pressure and cardiovascular risk control ○ Medication adherence counseling ○ Basic rehabilitation exercises and mobility support
Day 4	Patient Education and Communication Skills	○ Effective patient and family counseling ○ Lifestyle modification education (diet, exercise, smoking cessation) ○ Health promotion strategies in the community
Day 5	Integration and Case-Based Practice	○ Case studies covering prevention, recognition, referral, and follow-up ○ Role-play and skills demonstration ○ Group discussion on challenges and solutions in PHC settings

**Table 2:** Knowledge, attitudes, and practices of registered nurses towards stroke.

Variable	No.	KAP Questions	Answers	Pretest Score, n (%)	Posttest Score, n (%)	*p*-Value
knowledge	1.	Which of the following is the strongest independent risk factor for ischemic stroke?	(a) Diabetes mellitus (b) Hypertension √ (c) Dyslipidemia (d) Smoking	12 (34.1)	34 (100)	0.0001
	2.	The acronym FAST is used for stroke screening. What does it stand for?	(a) Facial droop, Arm weakness, Speech disturbance, Time to act √ (b) Facial droop, Alertness, Strength, Temperature (c) Fatigue, Arm movement, Speech, Treatment (d) Facial expression, Arm reflex, Sensation, Tremors	0	32 (93.3)	0.0001
	3.	A registered nurse is caring for a patient who presented 2 h after sudden onset of unilateral weakness and slurred speech. Which intervention is the most time-critical?	(a) Monitoring blood pressure (b) Keeping the patient nil per os (NPO) (c) Immediate referral for brain imaging (CT scan) √ (d) Administering IV fluids	12 (34.1)	31 (92.5)	0.0001
	4.	Which of the following correctly differentiates ischemic from hemorrhagic stroke?	(a) Ischemic stroke is associated with clot formation; hemorrhagic stroke results from vessel rupture √ (b) Ischemic stroke occurs only in older adults; hemorrhagic stroke only in young adults (c) Ischemic stroke presents with headache; hemorrhagic stroke does not (d) Ischemic stroke requires surgery; hemorrhagic stroke does not	3 (8.3)	34 (100)	0.0001
	5.	In the acute management of ischemic stroke, intravenous thrombolysis (tPA) should ideally be administered within:	(a) 1 h of symptom onset (b) 3–4.5 h of symptom onset √ (c) 6–12 h of symptom onset (d) 24 h of symptom onset	7 (20.8)	34 (100)	0.0001
	6.	The most appropriate initial diagnostic test to differentiate between ischemic and hemorrhagic stroke is:	(a) MRI of the brain (b) CT scan of the brain without contrast √ (c) Lumbar puncture (d) Electroencephalogram (EEG)	3 (8.3)	33 (96.6)	0.0001
	7.	Which of the following cardiac conditions is most strongly associated with embolic stroke?	(a) Atrial fibrillation √ (b) Myocardial infarction (c) Heart failure (d) Valvular regurgitation	3 (8.3)	34 (100)	0.0001
	8.	In the acute phase of hemorrhagic stroke, the nurse should anticipate which priority management?	(a) Anticoagulation therapy (b) Blood pressure control and prevention of rebleeding √ (c) Administration of thrombolytics (d) Immediate physiotherapy exercises	23 (66.6)	27 (80)	0.0001
	9.	The most common site of cerebral infarction in ischemic stroke is:	(a) Middle cerebral artery (MCA) territory √ (b) Posterior cerebral artery (PCA) territory (c) Anterior cerebral artery (ACA) territory (d) Basilar artery territory	23 (66.6)	34 (100)	0.0001
	10.	Which electrolyte imbalance may worsen cerebral edema and should therefore be closely monitored in acute stroke patients?	(a) Hyperkalemia (b) Hyponatremia √ (c) Hypocalcemia (d) Hypermagnesemia	3 (8.3)	34 (100)	0.0001
	11.	Which clinical scale is most commonly used in emergency settings to assess the severity of a stroke on admission?	(a) Glasgow Coma Scale (GCS) (b) National Institutes of Health Stroke Scale (NIHSS) √ (c) Modified Rankin Scale (mRS) (d) Barthel Index	2 (5)	34 (100)	0.0001
Attitudes	12.	Early recognition and rapid referral of stroke patients is primarily:	(a) The responsibility of doctors (b) A shared responsibility between nurses and the healthcare team √ (c) The family’s responsibility (d) Not a nurse’s role	31 (90)	34 (100)	0.0001
	13.	If a community member reports sudden facial drooping and slurred speech, what would be your immediate attitude towards this case?	(a) Treat as a medical emergency √ (b) Advise them to rest at home (c) Wait to see if symptoms resolve	25 (73.3)	32 (93.3)	0.0001
	14.	Stroke rehabilitation should begin:	(a) Only after hospital discharge (b) As early as possible during hospital admission √ (c) Only when requested by the patient’s family	3 (8.3)	34 (100)	0.0001
	15.	Do you consider it part of your professional duty as a nurse to educate stroke patients and their families about lifestyle modification?	(a) Yes √ (b) No	7 (20.8)	34 (100)	0.0001
	16.	Do you believe stroke is largely preventable through risk factor control (e.g., hypertension, diabetes, smoking)?	(a) Yes √ (b) No	3 (8.3)	33 (96.6)	0.0001
	17.	Providing training for village health workers on stroke recognition and prevention is:	(a) Beyond the nurse’s professional role (b) Part of the nurse’s professional responsibility √	23 (66.6)	33 (98.3)	0.0001
Practices	18.	A patient with acute stroke is admitted to your ward. What is your first nursing priority?	(a) Establishing airway patency and monitoring vital signs √ (b) Starting IV fluids (c) Administering antihypertensives immediately (d) Positioning the patient for comfort	0	34 (100)	0.0001
	19.	Before allowing oral intake in a stroke patient, the nurse should first:	(a) Check blood pressure (b) Conduct a swallowing assessment √ (c) Administer antiemetics (d) Start physiotherapy	25 (73.3)	32 (93.3)	0.0001
	20.	To prevent deep vein thrombosis (DVT) in an immobile stroke patient, the nurse should:	(a) Encourage early mobilization and use compression stockings if available √ (b) Maintain strict bed rest (c) Increase fluid intake only (d) Use sedatives to reduce agitation	7 (20.8)	34 (100)	0.0001
	21.	A nurse providing secondary prevention for a stroke survivor should focus on:	(a) Blood pressure control, lipid management, and adherence to antiplatelets √ (b) Only physiotherapy exercises (c) Family counseling alone (d) Reducing hospital visits	3 (8.3)	33 (96.6)	0.0001
	22.	In providing comprehensive stroke care, the nurse’s role includes:	(a) Acute management, rehabilitation support, and community health education √ (b) Only hospital-based care (c) Only rehabilitation support (d) Only physician assistance	23 (66.6)	33 (98.3)	0.0001

√—Correct answer.

**Table 3:** Biographical data of participants.

Variables	Categories	F(n)	Percent
Age	20–25 years	0	0
	26–30 years	2	6
	31–40 years	18	53
	41–50 years	12	35
	51 and above	2	6
N		34	100
Gender	Male	12	35
	Female	22	65
N		34	100
Qualification	Diploma in general nursing and midwifery	28	82
	Degree in nursing with nursing specialty	6	18
N		34	100
Work experience	6–12 months	0	0
	1–5 years	1	3
	6–10 years	10	29
	11–20 years	20	59
	20 or more years	3	9
N		34	100

**Table 4:** Effect of intervention on nurses’ knowledge, attitudes and practices.

Variable	Pre-Test Mean	Post-Test Mean	Cohen’s d	Interpretation
Knowledge	23.7%	96.6%	2.35	Very large effect
Attitudes	44.6%	98.0%	2.51	Very large effect
Practices	33.8%	97.6%	2.99	Very large effect

## Data Availability

The data presented in this study are available on request from the corresponding author (the data are not publicly available due to privacy restrictions).

## Public Involvement Statement

No public involvement in any aspect of this research.

## Guidelines and Standards Statement

This study confirms compliance with the Declaration of Helsinki and has received ethical approval from Lesotho’s Ministry of Health (ID 288-2024) National Health Research and Ethics Committee. The researchers conducted the study competently, following rigorous and methodologically sound procedures.
